# Fine-Needle Aspiration Cytology Can Play a Role in Neoadjuvant Chemotherapy in Operable Breast Cancer

**DOI:** 10.1155/2013/935796

**Published:** 2013-07-10

**Authors:** Christian Garbar, Hervé Curé

**Affiliations:** Departments of Biopathology and Medical Oncology, Institut Jean-Godinot-Unicancer, 51726 Reims Cedex, France

## Abstract

Despite the fact that CNB has been progressively replaced by FNAC in the investigation of nonpalpable lesions or microcalcifications without a clinical or radiological mass lesion, FNAC has yet a role in palpable lesions provided it is associated with the triple diagnosis and experienced cytologist. In these conditions, FNAC is a safe, effective, economical, and accurate technique for breast cancer evaluation. Numerous literature reviews and meta-analyses illustrated the advantages and disadvantages of both methods CNB and FNAC. The difference does not seem significant when noninformative and unsatisfactory FNAC was excluded. Recently, cytological methods using liquid-based cytology (LBC) technology improve immunocytological and molecular tests with the same efficiency as classical immunohistochemistry. The indications of FNAC were, for palpable lesions, relative contraindication of CNB (elderly or frailty), staging of multiple nodules in conjunction or not with CNB, staging of lymph node status, newly appearing lesion in patient under neoadjuvant treatment, decreasing of anxiety with a rapid diagnosis, evaluation of biomarkers and new biomarkers, and chronological evaluation of biomarker following the neoadjuvant therapy response.

## 1. Introduction

Neoadjuvant chemotherapy actually takes an important place in treatment of operable breast cancer in the hope of improving conservative surgery rate of female patients. Neoadjuvant chemotherapy includes today on target therapies involving the research on expression of specific molecules by tumoral cells. In routine practice, estrogen receptors (ER), progesterone receptors (PR), and human epidermal growth factor receptor 2 (HER2) are the common used biomarkers. New pharmaceutical molecules, other than ER/PR or HER2, are now already evaluated in clinical research as new biomarkers and new target for neoadjuvant therapy [1].

Fine-needle aspiration cytology (FNAC) of the breast is wellknown as a safe, effective, economical, and accurate technique for diagnosing palpable breast lesion [2–4]. This last decade, FNAC technique is improved by the development of new cytological methods allowing standardization of fixation and assuring constant results with ancillary tests such as immunocytochemistry and in situ molecular biology. Also, one of the advantages of FNAC is the management of small tissue fragments permitting a repetitive evaluation of the chronological evolution in expression of tumoral biomarkers. 

## 2. What Are the Advantages of FNAC in Comparison with Core-Needle Biopsy (CNB)?

Previously, the role of FNAC has been challenged by results obtained with CNB that seems more robust than FNAC. In general, CNB is now preferred in the first line of diagnosis [5]. Nevertheless, CNB carries disadvantage in terms of a long tissue processing time and patient discomfort such as pain (1.7% to 3.7%), hematoma (0.72%), and very rarely pneumothorax [6, 7]. FNAC includes more advantages than CNB such as minimal invasiveness and minimal discomfort (more painless) that could be interesting for aged or frailty patients with comorbidities [8]. In palpable lesions, FNAC is also easy to perform by nonradiologists as clinicians or pathologists. FNAC could perform repetitively and is a serious candidate for the chronological followup of neoadjuvant chemotherapy response.

An important quality of FNAC is its ability to give rapid diagnostic information equivalent to that of frozen sections [9]. In our experience, the result of rapid FNAC prior to CNB improves the quality of CNB and gives an immediate diagnosis decreasing anxiety of patient. 

Other indications of FNAC are staging of multiple tumors or suspicious zones and apparition of a new suspicious lesion during neoadjuvant chemotherapy. Finally, FNAC could be an excellent alternative when radiographic screening of breast is not available [9].  Table 1 compares the main advantages and disadvantages of FNAC versus CNB.

*Other Clinical FNAC Indications.*

- Palpable breast lesion.
- Rapid diagnosis to decrease anxiety of patient.
- Patient with morbidity (senile, cardiac, diabetic etc.).
- Clinical staging: multiple lesions, suspicious lymph node, and so on.
- Apparition of new lesion in patient treated for breast cancer.
- Evaluation of biomarkers.
- Evaluation of biomarker changes following time or metastasis.
- When CNB technique is not available.

## 3. Is the Diagnosis of FNAC Accurate?

It is well known that the combination of clinical evaluation, mammography, and FNAC, called triple diagnosis, gives a precise diagnosis [10, 11]. Yu et al. [12] recently demonstrated in meta-analysis of 46 studies that FNAC had a sensitivity of 92.7% and a specificity of 94.8% except the unsatisfactory samples. The ROC curve showed an excellent area under the curve of 0.986, presenting a high level of accuracy. On the other hand, if the FNAC result was negative, the probability of breast cancer is approximately 8%. These authors concluded that FNAC was an accurate material for evaluation on breast malignancy if rigorous criteria are used. Also, they said that FNAC may provide a favourable screening method and permit an improvement of treatment planning. Therefore, when FNAC is unsatisfactory, CNB is required to minimize the probability of a missed malignant diagnosis. In a study of FNAC and immediate diagnosis performed in 408 palpable breast lesions, Liew et al. in 2010 [9] reached the same conclusions: 98.1% sensitivity, 89.5% specificity, and 95.8% accuracy. In 508 CNB followed by Jackman et al. [13], the rate of false negative for all lesions was 4.4%, for microcalcifications alone 1.2%, and for tumoral mass 0.8%. These results of CNB are quasi-identical to those obtained with the satisfactory FNAC. Most of false negative FNAC results of sampling error or discordance between clinical and histological observations [6, 14]. In a comparison between CNB and repeat FNAC after an indeterminate diagnosis with FNAC, Kooistra et al. [15] suggested that CNB should be performed after an indeterminate FNAC to obtain a reliable preoperative diagnosis.

Other authors concluded that, although the FNAC is easier to perform, this technique was not efficient for small and nonpalpable lesions or diagnosis of microcalcifications as those for in situ carcinoma. These comments are likely true because some preneoplastic lesions are associated with slight cell atypia. However, Bilous [6] emphasized that CNB shows also problems with similar lesions such as atypical proliferative lesions (atypical ductal hyperplasia, in situ lobular neoplasia, etc.), cellular fibroepithelial lesions, papillary tumors, mucinous carcinoma, radial scar, spindle cell lesions. Moreover, FNAC interpretation requires serious experience of the cytopathologist and they think that this is one of the main reasons that overall CNB is to be preferred [16]. 

A recent Japanese study [17] of 5693 FNAC and 7 different laboratories illustrated a great variability between the institutions suggesting likely difference between education of cytologists and the lack of clinical or radiological information (triple diagnosis).

## 4. What about Biomarkers?

These last years a new cytological technique, called liquid-based cytology (LBC), has been developed and approved by the Food and Drug Administration. Briefly, LBC standardises the cell fixation, concentrates epithelial cells, and discards blood cells and/or cell debris that obscure the smear. The lecture of LBC seems therefore easier than that of conventional smear. The efficiency of LBC in the breast cytology has been demonstrated by numerous publications. 

The main advantage of LBC is surely to adjunct ancillary tests such as immunocytochemistry, flow cytometry, or molecular biology [18–20]. Domanski et al. [21] compared the ER and PR statuses from FNAC (immunocytochemistry) and CNB (immunohistochemistry), both performed on surgical breast tumors. They found that both methods give similar results with a concordance between the 2 tests of 98% for ER (with kappa correlation score = 0.93) and 96% for PR (kappa = 0.91). Monaco et al. [22] demonstrated similar data in a comparative study between primary breast tumours and their metastasis. Interestingly, the concordance between these both localisations was 81% for ER, 65% for PR, and 71% for HER2, suggesting a possibility of biological difference between primary tumors and their metastasis. 

Other publications showed a long-time storage at −20°C and −80°C at least 6 months without significant loss of immunoreactivity of PR and EP from breast FNAC [23]. This suggested that tumor cell bank is feasible. 

**Figure 1:**
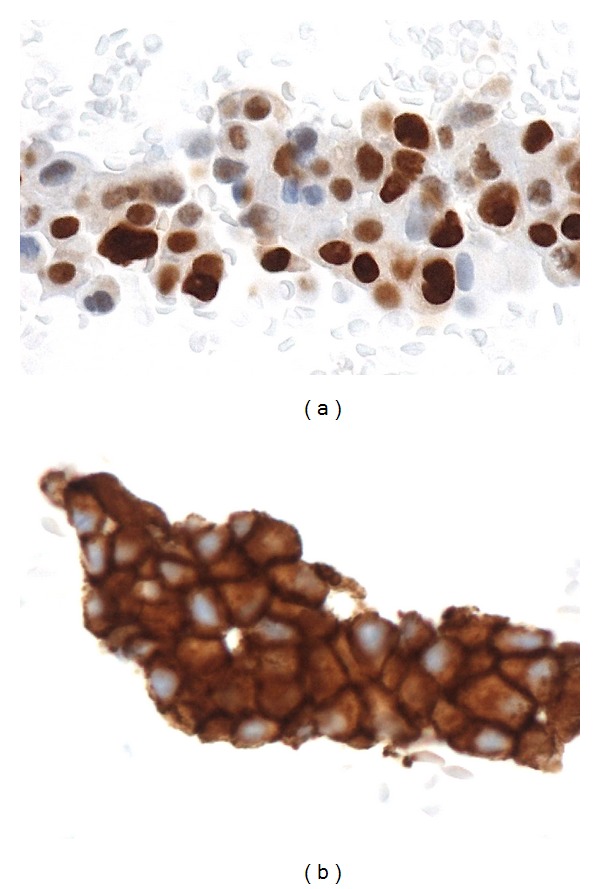
FNAC immunocytochemistry estrogen. receptors (a) and HER2 (b).

**Figure 2:**
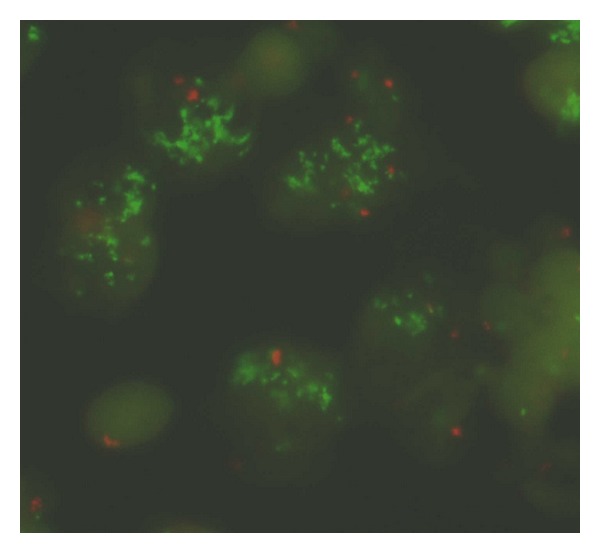
In situ molecular biology. FISH: amplification of HER2 gene (green spots).

The cell block cytology is an attractive cytological method for ancillary techniques or long-time cell conservations and consists in putting cells of FNAC directly in formol fixative fluid identically at a classical histology (Figures 1 and 2: ER, HER2 and FISH on cell block cytology). Briefly, after centrifugation to concentrate cells, the pellet was embedded in a synthetic polymer gel that is then processed in paraffin block that could be cut at 4 *μ*m, as classical biopsy slides. With this technique, Ferguson et al. [24] found a concordance rate of 95% for ER, 90% for PR, and 88% for HER2. Similar results were observed by Shabaik et al. [25] with high specificity (100% for both) and lower sensibility (85% and 80% resp. for ER and PR). Finally, in FNAC, false negative ER or PR immunostaining exists but false positive tests are very unlikely. False negative immunohistochemical results are also observed in CNB: in a retrospective study, Seferina et al. [26] calculated a rate of false negative of 26.5% and a rate of false positive of 63.8% for both ER and PR. For HER2, they showed 5.4% for false negative rate and 50% for false positive rate. This discordance is likely explained by the heterogeneity of large tumors [6]. Nevertheless, in our experience, this discordance is often associated with the manipulation of CNB before the fixation (crush artefacts) or with a defect of fixation as desiccation. Technically, these mismanipulations do not exist with LBC. 

Fortunately the concordance with molecular biology by hybridisation in situ using FISH, CISH, and SISH is very good and can help when HER2 is uncertain [6, 25, 27]. The FISH is accurate for LBC cytology [22, 25, 28, 29]. The extraction of mRNA or DNA is also feasible from LBC and FNAC, allowing all gene expression analyses [29–32]. In our experience, FISH slides using the cell block method are easier to read.

## 5. Sentinel Lymph Node Evaluation by FNAC

The clinical staging and preoperative lymph node status are important for the evaluation of eligible patient to neoadjuvant therapy. In the axillary lymph node FNAC, Chang et al. [33] calculate 88.0% sensitivity and 97.8% specificity in a study on 163 women. Similar results were published by Oz et al. [34]. 

FNAC in lymph node is a cost effective and safe method, false positive is virtually non-existent, and false negative can occur when lymph node is partially involved such as by micrometastase, or isolated tumor cells [35]. 

In our experience, we improve axillary lymph node FNAC/LBC by immunocytochemistry using cytokeratin antibody. 

Thus, axillary FNAC plays a role in staging of advanced cases for systemic and neoadjuvant therapy and in evaluating candidates for sentinel lymph node surgical procedure or axillary lymph node dissection.

## 6. Conclusions 

Despite the fact that CNB has been progressively replaced by FNAC in the investigation of nonpalpable lesions or microcalcifications without a clinical or radiological mass lesion, FNAC has yet a role in palpable lesions in the triple diagnosis association and performed by experienced cytologists. In these conditions, FNAC is a safe, effective, economical, and accurate technique for breast cancer evaluation. 

Recently, cytological methods using LBC technology, associated or not with the cellblock cytological technique, improve immunocytological and molecular tests with the same efficiency as classical histology. 

If the limits of its indications are well known, FNAC still plays a role in the modern oncological practice. 

## Figures and Tables

**Table 1:** Advantages and disadvantages of FNAC versus CNB.

	FNAC	CNB
*General considerations *		
Rapid diagnosis	Yes	No
Special experience required	Yes	No
Pain discomfort	Very low	Low
Complication rate	Very low	Low
*Diagnostic performances *		
Accurate for nonpalpable lesions or microcalcifications	No	Yes
Accurate for palpable lesions or mass with microcalcifications	Yes	Yes
Distinction between in situ and invasive carcinoma	No	Yes
Distinction of low grade lesions (ADH, papilloma, etc.)	Very difficult	Difficult
Unsatisfactory sample	High	Low
Immunohistochemistry	Yes	Yes
In situ hybridisation	Yes	Yes
DNA/RNA isolation for molecular biology	Yes	Yes
Standardization of fixation	Very optimal	Optimal
Tissue/cell bank	Yes	Yes
